# Insights into the Bioactivities and Mechanism of Action of the Microbial Diketopiperazine Cyclic Dipeptide Cyclo(L-leucyl-L-prolyl)

**DOI:** 10.3390/md23100397

**Published:** 2025-10-09

**Authors:** Christian Bailly

**Affiliations:** 1 UMR9020-U1277-CANTHER-Cancer Heterogeneity Plasticity and Resistance to Therapies, CHU Lille, CNRS, Inserm, OncoLille Institute, University of Lille, 59000 Lille, France; christian.bailly@univ-lille.fr; 2 Institute of Pharmaceutical Chemistry Albert Lespagnol (ICPAL), Faculty of Pharmacy, University of Lille, 59006 Lille, France; 3 OncoWitan, 59290 Lille, France

**Keywords:** cyclo(Leu-Pro), diketopiperazine, gancidin, microorganisms, mechanism of action

## Abstract

Diketopiperazines (DKPs) are biologically important cyclic dipeptides widespread in nature, associated primarily with microorganisms. This is the case for the 2,5-DKP derivative cyclo(L-Leu-L-Pro) (cLP), also known as gancidin W or PPDHMP, identified from a variety of bacteria and fungi, and occasionally found in food products. The present review retraces the discovery of cLP, its identification in living species, its chemical syntheses, and its biochemical properties. In bacteria, cLP is often associated with other DKPs to serve as a defense element against other microorganisms and/or as a regulator of bacterial growth. cLP plays a role in quorum-sensing and functions as an anticariogenic and antifungal agent. The antimicrobial mechanism of action and molecular targets of cLP are evoked. The interest in cLP for combatting certain parasitic diseases, such as malaria, and cancers is discussed. The capacity of cLP to interact with CD151 and to down-regulate the expression of this tetraspanin can be exploited to reduce tumor dissemination and metastases. The review sheds light on the pharmacology and specific properties of cyclo(L-Leu-L-Pro), which can be useful for the development of a novel therapeutic approach for different human pathologies. It is also of interest to help define the bioactivity and mechanisms of action of closely related DKP-based natural products.

## 1. Introduction

**Figure 1:**
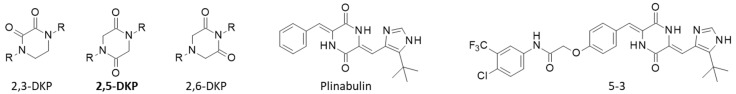
Diketopiperazine (DKP) motifs and structures of plinabulin and its derivative 5-3.

The diketopiperazine (DKP) motif is frequently encountered in drugs and natural products. There are three DKP isomers: 2,3-, 2,5-, and 2,6-DKPs (Figure 1). The 2,5-DKP motif is the one most commonly found in natural products, but the two other configurations are also exploited for drug design. For example, a recent work explored 2,3-DKP as a potential scaffold for developing antiparasitic compounds to combat Chagas disease [1]. The motif can be encountered occasionally in natural products, such as the alkaloids heterpyrazines A-B [2] and orychophragvioline A [3]. The 2,6-DKP unit is not very common, but it has been explored for designing anticonvulsant, anticancer, and antiparasitic compounds, for example [4,5,6,7,8,9]. In contrast, 2,5-DKPs are largely represented in nature, as observed in various alkaloids such as brevianamides E1-E2, amauromine, naseseazines, and many other natural products [10,11]. The drug candidate plinabulin is a 2,5-DKP derivative acting as an anti-tubulin agent, currently undergoing phase 3 clinical trials for the treatment of non-small-cell lung cancer [12,13], and its derivative 5-3 has revealed a promising antileukemic activity [14] (Figure 1). The DKP motif is considered important to the design of anticancer agents [15].

**Figure 2:**
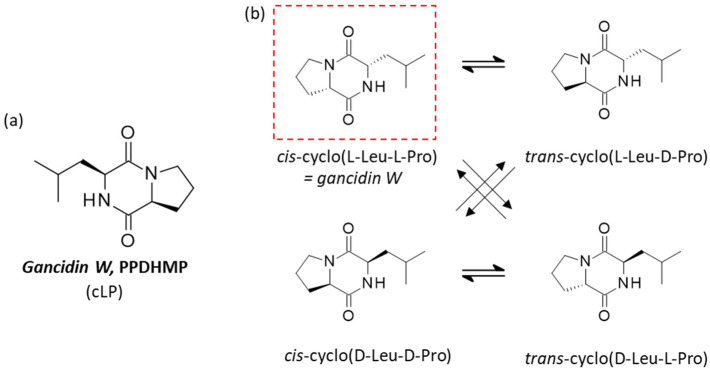
(**a**) Structure of *gancidin W* and (**b**) the different isomers of cyclo(Leu-Pro). *Gancidin W* corresponds to cis-cyclo(L-Leu-L-Pro) [cLP] or PPDHMP, (3S,8AR)-3-isobutylhexahydropyrrolo[1,2-a]pyrazine-1,4-dione.

DKP is a basic unit both in drug design and protein chemistry. The 2,5-DKP motif is in fact a cyclodipeptide unit obtained by the condensation of two α-amino acids. 2,5-DKP itself corresponds to cyclo(Gly-Gly), and its cis-amide functionality can form intermolecular hydrogen bonds (N−H...O) between adjacent molecules, so as to generate higher-ordered supermolecular structures in the solid state. This motif has been amply characterized, with multiple synthetic accesses to 2,5-DKPs proposed [16]. It is the simplest cyclic form of peptides, widespread in nature, resulting from the assembling of two amino acids by nonribosomal peptide synthetases or by cyclodipeptide synthases [17]. DKP dipeptides are endowed with diverse pharmacological properties, such as antimicrobial, insecticidal, antiviral, nematicidal activities, and others [18]. For example, cyclo(His-Pro) DKP isomers have shown activity against acetylcholinesterase and revealed neuroprotective properties of potential interest to combat Alzheimer’s disease [19]. In contrast, Trp-containing DKPs showed marked activities against human pathogenic bacteria [20], whereas the DKPs cyclo(His-Met) and cyclo(His-Pro) exhibited interesting anti-age properties [21]. Among the many existing DKP dipeptides, one natural product caught our attention: the leucine derivative cyclo(L-Leu-L-Pro), also known as gancidin W or PPDHMP, which displays interesting antimicrobial properties. An overview of the pharmacological properties of cyclo(L-Leu-L-Pro), hereafter designated cLP (Figure 2), is presented here, with the objective to promote knowledge on this atypical compound and to encourage the design of analogs.

## 2. cLP as a Natural Product

Gancidin A is an antibacterial natural product first isolated from a *Streptomyces gancidicus* strain AAK-84 in the mid-1950s at Chiba University, Japan [22]. It is a complex molecule, initially described with the formula C_43_H_58_N_6_O_14_, structurally related but larger than the quinoid antibiotic xanthomycin A (C_29_H_40_Cl_2_N_8_O_8_). The product has revealed an inhibitory activity against a wide range of pathogenic bacteria, in particular Gram-positive cocci [23]. The antibacterial effects have been reported but, from a structural standpoint, the antibiotic gancidin A has never been very well characterized [24,25]. In fact, the initial studies referred to three entities: gancidin complex, gancidin A, and gancidin W (C_11_H_18_N_2_O_2_), the former being 20 times more potent than the latter at inhibiting the growth of Ehrlich ascites carcinoma (MIC = 10 and 200 mcg/mL, gancidin A and W, respectively) [26]. If the structure of gancidin A remains unclear at present, that of gancidin W is well established; it corresponds to cis-cyclo(L-Leu-L-Pro), which is a compound originally known as L-Leucyl-L-Proline anhydride, produced by *Streptomyces* species [27,28,29]. Apparently, the compound was first discovered at the beginning of the XXth century from a tryptic digest of the gluten protein gliadin [30]. A product with the same formula (C_11_H_18_N_2_O_2_), designated helmintin, has been isolated from the deuteromycete *Helminthosporium siccans*, but it is also L-Leucyl-L-Proline anhydride or cLP [31] (Figure 2).

**Figure 3:**
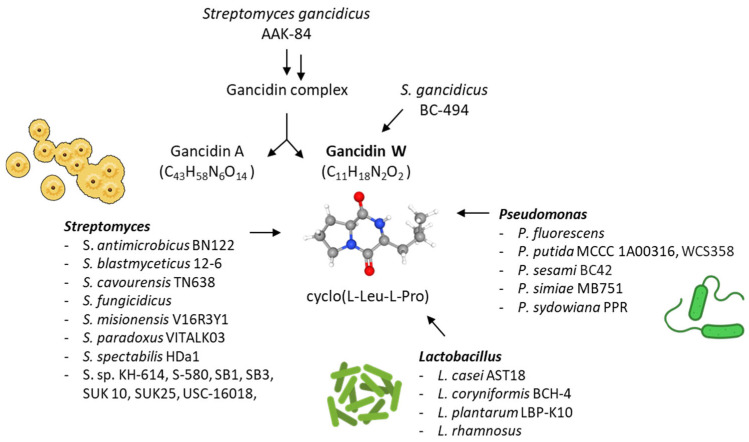
Microorganisms producing cLP (see Table 1 for more details).

In 1977, Jain and coworkers isolated and purified gancidin W from *Streptomyces gancidicus* strain BC-494 and structurally characterized the molecule using mass spectrometry and nuclear magnetic resonance (NMR) spectroscopy [32]. Over the past forty-eight years, gancidin W has been isolated from different microorganisms (Table 1). The compound has been found in the marine ascomycete *Corollospora pulchella* [33,34], the marine species *Streptomyces paradoxus* VITALK03 [35,36], *Streptomyces* species KH-614, SUK10, and VITLGK012 [37,38,39,40,41], and the endophytic fungal strain *Acremonium* sp. Ld-03 [42]. Gancidin W corresponds to the cis-cyclo(L-Leu-L-Pro) isomer (Figure 2). The other isomers also exist in nature. The two isomers cyclo(D-Leu-D-Pro) and cyclo(D-Leu-L-Pro) have been isolated from *Pseudonocardia endophytica* VUK-10 and characterized as antifungal agents [43]. Isomer cyclo(L-Leu-D-Pro) has been found in *Bacillus amyloliquefaciens* Y1 [44], but another study referred to the identification of cyclo(L-Leu-L-Pro) in this bacterium [45]. Isomers cyclo(L-Leu-D-Pro) and cLP have been isolated, together with many other DKPs, from a culture of the fungus *Phellinus igniarius* [46]. The present analysis is focused on the LL isomer only, found in many microorganisms (Figure 3).

cLP has been identified in bacterial fractions from the species *Staphylococcus xylosus* VITURAJ10 isolated from goat milk and shown to inhibit bacterial pathogens such as *S. aureus* and *E. coli*. In this case, the product was designated PPDHMP (for pyrrolo[1,2α]pyrazine-1,4-dione,hexahydro-3-(2-methylpropyl)) [47,48,49]. The same product PPDHMP has been isolated from a few other bacterial species, notably *Bacillus* sp. VITLTMJ4 [50], *Burkholderia seminalis* JRBHU6 [51], *Staphylococcus* sp. MB30 [52], and *Nocardiopsis* sp. GRG1 [53]. Actually, the denomination cyclo(L-Leu-L-Pro) is more frequently used than gancidin W or PPDHMP, but it is the same compound. Cyclo(L-Leu-L-Pro) has been found in the Gram-positive bacterium *Lactobacillus plantarum* LBP-K10 [54,55,56], Gram-negative bacterium *Pseudomonas sesami* BC42 [57], in extracts of *Streptomyces misionensis* V16R3Y1 [58], in *Lactobacillus coryniformis* BCH-4 [59], and in other microorganisms, including lactic acid bacteria present in Bulgarian yogurt, for example [60] (Table 1). Cyclo(Leu-Pro) displays marked antibacterial properties, with activities reported against both Gram-positive (*B. subtilis*, *S. aureus*) and -negative (*E. coli*, *S. typhimurium*) bacteria. Interestingly, the compound is also active against multidrug-resistant strains, such as *S. aureus* 11471 and *S. typhimurium* 12219c, with a level of activity significantly superior to that of the DKP cyclo(Phe-Pro) (MIC = 17.28 and 46.22 mg/mL, respectively, against *S. aureus* 11471) [61].

**Table 1:** Microorganisms producing cyclo(L-Leu-L-Pro) or gancidin W and associated bioactivities.

Microorganisms ^1^	Org. ^2^	Bioactivities	Refs
*Achromobacter xylosoxidans* NFRI-A1	B (g^−^)	Inhibition of fungal growth by cLP isolated from this endophytic bacterium. cLP repressed transcription of the aflatoxin-related genes.	[62]
*Aspergillus aculeatus* F027	F	Isolation of cyclo(L-Pro-L-Leu) and cyclo(L-Pro-L-Phe) and their antibacterial activities.	[63]
*Bacillus amyloliquefaciens* MMS-50	B (g^+^)	Characterization of cLP and its activity against *Streptococcus mutans*, responsible for dental caries.	[64]
		Antibiofilm activity of cLP against *Listeria monocytogenes* (MIC = 512 μg/mL).	[45]
*Bacillus baekryungensis* AMHSU	B (g^+^)	Isolation of PPDHMP (cLP) and characterization of its anti-inflammatory activity.	[65]
*Bacillus pumilus* GL0057	B (g^+^)	Two DKPs, cyclo-(L-Leu-L-Pro) and cyclo-(L-Phe-L-Pro), identified from this marine bacterium isolated from the black coral *Antipathes* sp.	[66]
*Bacillus* sp. strain N	B (g^+^)	Marked activity of cyclo(l-Pro-l-Leu) against the fungus pathogen *Penicillium expansum*.	[67]
*Bacillus* sp. VITLTMJ4.	B (g^+^)	Identification of PPDHMP (cLP) isolated from this bacterial species endophyte of *Citrus limon* (Kaji nemu) and its antibacterial properties.	[50]
*Bacillus vallismortis* BS07	B (g^+^)	Characterization of cyclic dipeptides, including cyclo(L-Leu-L-Pro), and their role in disease resistance in *Arabidopsis* against *Pseudomonas syringae* infection.	[68]
*Bacillus velezensis* Ea73	B (g^+^)	Cyclo(L-Leu-L-Pro) and cyclo(L-Pro-L-Val) extracted from this endophytic bacterium in the poisonous weed *Ageratina adenophora*.	[69]
*Brevibacillus laterosporus*	B (g^+^)	Identification of cyclo(Leu-Pro) and its activity against several pathogenic microorganisms.	[70]
*Corollospora pulchella*	F	Production of gancidin W by the marine fungus *C. pulchella*.	[33,34]
*Cronobacter sakazakii*	B (g^−^)	The role of cyclo(l-Pro-l-Leu) as a *quorum-sensing* signal between *C. sakazakii* and *Bacillus cereus*.	[71]
*Exiguobacterium acetylicum* S01	B (g^+^)	Four DKPs identified, including cLP, capable of inducing cell growth arrest and apoptosis of HT-29 cancer cells. The four DKPs inhibited tumor progression in a zebrafish xenograft model.	[72]
*Exiguobacterium* R2567	B (g^+^)	In this bacterium, cyclo(Leu-Pro) activates the rice strigolactone signaling pathway by binding to the SL receptor OsD14, so as to regulate tillering.	[73]
*Galactomyces geotrichum*	F	Identification of cyclo(Leu-Pro) as a metabolite in this species from *Laminaria japonica*.	[74]
*Haemophilus influenzae* Rd KW20	B (g^−^)	Identification of cyclo(Leu-Pro) as a metabolite in this species via a genome-scale metabolic model.	[75]
*Lactiplantibacillus plantarum* CCFM8724	B (g^+^)	Identification of cyclo(leu-pro) and cyclo(phe-pro) and their roles as biofilm inhibitors.	[76]
*Lactobacillus casei* AST18	B (g^+^)	Identification of cyclo(Leu-Pro) and its synergistic antifungal effect with lactic acid against *Penicillium* sp.	[77]
*Lactobacillus coryniformis* BCH-4 (*Loigolactobacillus coryniformis* BCH-4)	B (g^+^)	Identification of cLP and characterization of its antifungal action against *Aspergillus flavus* and potential target proteins. Bioprotective activity.	[59]
*Lactobacillus plantarum* LBP-K10	B (g^+^)	Identification of five DKPs, including cyclo(l-Leu-l-Pro), and their inhibitory effects against *Ganoderma boninense*.	[54,55]
		Antiviral activity of fractions containing *cis*-cyclo(L-Leu-L-Pro) against *Influenza* virus.	[56]
		Antimicrobial activity of cyclo(L-Leu-L-Pro) against multidrug-resistant bacteria, alone and in combination with a microbial fraction (Q9).	[78]
*Lactobacillus rhamnosus*	B (g^+^)	Identification of cyclo(L-Leu-L-Pro) and its antibiofilm activity.	[79]
*Lactococcus lactis* subsp. *cremoris*	B (g^+^)	Identification of cLP in this species.	[80]
*Lasiodiplodia iranensis* F0619	F	Identification of cLP in a fractionated extract of *L. iranensis* isolated from the Panamean mangrove *Avicennia germinans.*	[81]
*Leuconostoc mesenteroides* LBP-K06	B (g^+^)	Bacteria found in fermented food kimchi and characterization of cyclo(Leu-Pro) and its activity against different Gram-positive/negative bacteria.	[61]
		Optimization of culture conditions to produce DKPs in this system, notably with co-culture of *Lb. plantarum* LBP-K10 and *Leu. mesenteroides* LBP-K06.	[82]
*Limosilactobacillus reuteri* LR-9 (*Lactobacillus reuteri*)	B (g^+^)	Identification of cyclo(L-Pro-L-Leu), cyclo(L-Pro-L-Phe), and the antifungal activity of bacterial fractions.	[83]
*Lysobacter capsici* AZ78	B (g^−^)	Isolation of cyclo(l-Pro-l-Leu) and other DKPs, and their activity against Gram-positive bacterium *Rhodococcus fascians* LMG 3605.	[84]
*Marchantia polymorpha*	L	Identification of cLP and cyclo(l-Phe-l-Pro) produced by endophytes from *M. polymorpha.*	[85]
*Nocardia ignorata*	B (g^+^)	Identification of cyclo(l-Pro-l-Leu) and other DKPs from this actinobacterium isolated from the terrestrial lichen *Collema auriform*.	[86]
*Nocardiopsis* sp. GRG 1	B (g^+^)	Activity of a bacterial extract against biofilm forming uropathogens and identification of PPDHMP (cLP) from this species.	[53]
*Nocardiopsis* sp. HT88	B (g^+^)	Identification of 8 DKPs, including cyclo(*L*-Pro*-L*-Leu), from the endophytic bacterium of *Mallotus nudiflorus* L.	[87]
*Penicillium purpurogenum* G59	F	Different Pro-containing DKPs, including cLP, were isolated from a neomycin-resistant mutant of this marine-derived fungus, together with penicimutide.	[88]
*Pestalotiopsis sydowiana* PPR	F	cLP from the marine fungal *P. sydowiana* inhibits biofilm formation by *Pseudomonas aeruginosa* PAO1 at sub-toxic concentrations. Anti-QS activity at sub-MIC concentrations of cLP.	[89]
*Pithomyces sacchari*	F	cLP isolated with two other DKPs from the endophytic fungus *Pithomyces sacchari* of the *Laurencia* sp. collected in the South China sea.	[90]
*Pseudofusicoccum* sp.	F	Isolation and characterization of cyclo(L-Pro-L-Val) and cyclo(L-Leu-L-Pro) in this fungus, which produces the burgundy pigment upon fermentation.	[91]
*Pseudomonas fluorescens*	B (g^−^)	Isolation of cyclo(L-Leu-L-Pro) and bactericidal activity against *S. aureus* and *P*. *aeruginosa.*	[92]
*Pseudomonas putida* MCCC 1A00316	B (g^−^)	Identification of cyclo(l-Pro-l-Leu) and characterization of its activity against the nematode *Meloidogyne incognita*. The product increased the mortality rates of second-stage juveniles (J2) of *M. incognita*.	[93]
*Pseudomonas putida* WCS358	B (g^−^)	Identification of four DKPs, including cyclo(l-Leu-l-Pro), and characterization of their capacity to activate the quorum-sensing biosensors of the plant pathogen *Agrobacterium tumefaciens*.	[94]
*Pseudomonas sesami* BC42	B (g^−^)	Identification of three isomers of cyclo(Leu-Pro), including cyclo(l-Leu-l-Pro), which potently reduced conidia germination and leaf lesion size caused by the fungal pathogen *Colletotrichum orbiculare*.	[95]
*Pseudomonas simiae* MB751	B (g^−^)	Isolation of cyclo(L-Pro-L-Leu) from this nematicidal bacterium and its capacity to kill the root-knot nematode *Meloidogyne incognita*.	[96]
*Pseudomonas* sp. PTR-08	B (g^−^)	Antioxidant and anti-glycation activities of the bacterial extract that contains PPDHMP (cLP).	[97]
*Rheinheimera japonica* KMM 9513^T^	B (g^−^)	cLP and other DKPs identified from this marine bacterium. No antibacterial activity observed with cLP.	[98]
*Rosellinia necatrix*	F	Identification of three DKPs, including cyclo(Leu-Pro), and their capacity to inhibit the growth of plant seedlings and plant roots.	[99]
*Ruegeria* sp.	B (g^−^)	Identification and structural characterization of cyclo(Leu-Pro) in an extract of *Ruegeria* sp. from Indonesia.	[100]
*Sceloporus virgatus* (lizard)	A	Identification of cyclo(L-Leu-L-Pro) and cyclo(L-Pro-L-Pro) in the femoral gland secretions of the lizard.	[101]
*Shewanella baltica* SA02	B (g^−^)	Production of cyclic dipeptides, including cyclo(L-Pro-L-Leu), and their role in the production of biofilm matrixes.	[102]
*Staphylococcus xylosus* VITURAJ10	B (g^+^)	Isolation of PPDHMP (cLP) from the strain VITURAJ10 isolated from goat milk and antimicrobial activity of the bacterial extract.	[46]
*Staphylococcus* sp. MB30	B (g^+^)	Isolation of PPDHMP from this marine bacterium and characterization of its antiproliferative and pro-apoptotic properties using lung (A549) and cervical (HeLa) cancer cells.	[52]
*Streptomyces antimicrobicus* BN122.	B (g^+^)	Cyclo-(L-Pro-L-Xxx) DKPs, including cLP, identified from the *Streptomyces* strain, which is an endophyte in *Oryza sativa*.	[103]
*Streptomyces blastmyceticus* 12-6	B (g^+^)	Identification of cyclo-(Leu-Pro) and characterization of its activity against several pathogenic fungi, notably the spores of *Colletotrichum acutatum* responsible for anthracnose in plants.	[104]
*Streptomyces cavourensis* TN638	B (g^+^)	cLP identified as one of the DKPs present in the studied extracts, and its antibacterial activities.	[105]
*Streptomyces fungicidicus*	B (g^+^)	Isolation of cyclo(l-Leu-l-Pro) and four other DKPs from a culture of this deep-sea actinomycete bacterium, and their activities against the larvae of the barnacle *Balanus amphitrite*.	[106]
*Streptomyces gancidicus* BC-494	B (g^+^)	The first strain from which gancidin W (cLP) was identified and structurally characterized.	[32]
*Streptomyces griseorubens* K5	B (g^+^)	Metabolite profiling of the bacterial extract and identification of PPDHMP (cLP).	[107]
*Streptomyces lavendulae* No. 314.	B (g^+^)	Isolation of Pro-containing DKP, including cLP, from a culture filtrate of this species.	[108]
*Streptomyces misionensis* V16R3Y1	B (g^+^)	Identification of cyclo(l-Leu-l-Pro) in fractions active against human pathogenic bacteria.	[58]
*Streptomyces paradoxus* VITALK03	B (g^+^)	Production of gancidin W by this marine strain. Evaluation of its cytotoxic properties (IC_50_ = 1.56 μg/mL against MCF7 breast cancer cells) and potential binding to protein targets using molecular modeling (binding to Kras).	[35,36]
*Streptomyces* sp. KH-614	B (g^+^)	Marked activity against vancomycin-resistant enterococci, notably *E. faecalis* (strains K-99-34, K-00-184, and K-00-221); MIC = 12.5 m g/mL.	[37]
		Potent activity of cyclo(l-Leu-l-Pro) against the phytopathogenic fungus *Pyricularia oryzae* IFO5994 (MIC = 2.5 mg/mL).	[109]
		Cyclo(l-Leu-l-Pro) inhibits the growth of different pathogenic microorganisms and displays anti-mutagenic effects in *Salmonella* strains.	[110]
*Streptomyces* sp. S2A	B (g^+^)	Antibacterial activities of extracts from this species and characterization of PPDHMP (cLP) as a main bioactive product.	[111]
*Streptomyces* sp. S-580	B (g^+^)	Isolation of l-Leucyl-l-Proline anhydride and the formation mechanism of l-prolyl diketopiperazines.	[29,112,113]
*Streptomyces* sp. SB1 and SB3	B (g^+^)	Identification of cLP and other DKPs produced by *Streptomyces* species SB1 and SB3.	[114]
*Streptomyces* sp. SUK 10	B (g^+^)	Gancidin W was produced by bacteria *Streptomyces*, sp. SUK10, identified from the bark of the *Shorea ovalis* tree. The dipeptide was shown to inhibit the growth of *Plasmodium berghei* PZZ1/100 in mice.	[38,39]
*Streptomyces* sp. SUK 25	B (g^+^)	Identification of five DKPs, including cLP, and their activities against methicillin-resistant *S. aureus* and *Enterococcus raffinosus*.	[115]
*Streptomyces* sp. USC-16018	B (g^+^)	Antiplasmodial activity of cyclo(l-Pro-l-Leu), but not cyclo(l-Pro-l-Phe), cyclo(l-Pro-l-Val), and cyclo(l-Pro-l-Ty), against *Plasmodium falciparum* strains 3D7 and Dd2, without cytotoxicity.	[116]
*Streptomyces* sp. VITMK1	B (g^+^)	Isolation of PPDHMP (cLP) and characterization of its free radical scavenging activity.	[117]
*Streptomyces spectabilis* HDa1	B (g^+^)	Isolation of three DKPs, including cyclo-(L-Leu-l-Pro). No acetylcholinesterase inhibitory activity observed with this DKP.	[118]
*Veillonella tobetsuensis*	B (g^−^)	Characterization of cLP and its capacity to inhibit *Streptococcus gordonii* biofilm development.	[119]

^1^ Main examples. Cyclo(Leu-Pro) has been found in other species, but the stereochemistry of the product is not always indicated. cLP = cyclo(Leu-Pro). ^2^ Organisms: A, animal; B, bacteria (g^−/+^, Gram^negative/positive^); F, fungus; L, liverwort.

Altogether, cLP has been found in a large variety of microorganisms, including *Streptomyces, Lactobacillus*, and *Pseudomonas* species (Table 1). But, remarkably, the product is not exclusive to microorganisms. It has been found also in a few plants and in terrestrial vertebrates, notably in the striped plateau lizard (*Sceloporus virgatus*). Two DKPs, cyclo(L-Leu-L-Pro) and cyclo(L-Pro-L-Pro), were found in the femoral gland secretions of the lizard and were suspected to play a role in intra-specific (male–male) communication of lizards [101]. DKPs have been found in beef, including cis-cyclo(L-Leu-L-Pro), contributing to the organoleptic properties [120].

In plants, cLP was identified in a bioactive fraction prepared from an aqueous extract from the medicinal plant *Fagonia cretica*. This fraction showed activity against multidrug-resistant gastrointestinal pathogenic bacteria [121]. The presence of cLP in the orchid *Gymnadenia conopsea* (L.) R. Br.—an endangered medicinal plant—has been reported as well [122]. The cyclic dipeptide has been found in food products, such as Bulgarian yogurts as mentioned above [60], but also in beer, soy sauce, and roasted coffee [123,124,125]. cLP was identified in the millet-based fermented beverage tongba, consumed by Nepalese–Tibetan communities in Himalaya [126,127]. It can even be found in bread crumbs and crusts, together with cis-cyclo(L-Phe-L-Pro), formed during the baking process [128]. cLP or gancidin W is a natural product largely present in nature, essentially produced by microorganisms, but occasionally also by plants and vertebrates.

The biosynthetic pathway leading to cLP is not well defined. Like other DKPs, the initial dipeptide can be formed through protein degradation or enzymatic pathways [129]. DKP biosynthesis pathways are complex. A recent study pointed out the existence of 359 cyclodipeptide synthases (CDPSs) and 9482 nonribosomal peptide synthetases (NRPSs) responsible for DKP biosynthesis in fungi [130]. In *Pseudomonas aeruginosa*, non-ribosomal peptide synthase (NRPS) proteins are implicated in the biosynthesis of these cyclodipeptides, notably cLP [131].

## 3. Chemical Synthesis of cLP

**Scheme 1:**
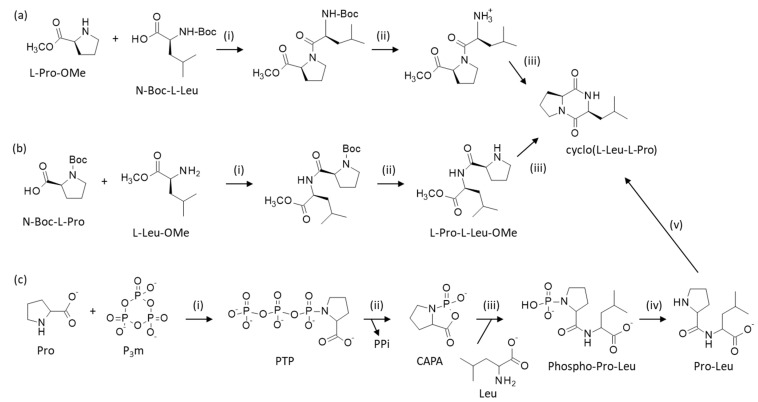
Three complementary strategies for the synthesis of cLP. (**a**) Synthetic pathway used to prepare a DKP library including cLP. Conditions: (i) EDC-HCl, Et_3_N, CH_2_Cl_2_, 4 °C, 16 h. (ii) AcCl, MeOH, 0 °C, 3 h. (iii) Piperidine, DMF, 25 °C, 1 h [133]. (**b**) Synthesis based on the coupling of N-Boc-Pro with Leu-OMe to obtain the dipeptide, then cyclized after Boc-deprotection with TFA. (i) EDC, HOBt, Et_3_N, CH_2_Cl_2_, r.t. 24 h. (ii) CF_3_COOH, r.t., 1h. (iii) Et_3_N, CH_3_OH, reflux, 2 h [135]. (**c**) Synthesis of cyclo(Leu-Pro) according to [137]. (i) Pro activation with Na trimetaphosphate (P3m) (NaOH,H_2_O). (ii) formation of the cyclic acylphosphoramidate (CAPA) intermediate, which is then (iii) coupled with Leu to obtain phopho-Leu-Pro. After deprotection (iv), the final product was obtained upon cyclization in the alkaline aqueous solution (v).

Most biologically active DKPs are isolated from natural sources, but these products can be obtained via synthetic methods, starting from α-amino acids, followed by the formation of a dipeptide and a cyclization [132]. The four stereoisomers of cyclo(Leu-Pro) can be easily prepared through chemical synthesis via the coupling of N-Boc protected leucine (N-Boc-Leu) with proline methyl ester (Pro-OMe), or via the reverse situation using N-Boc protected proline (N-Boc-Pro) and leucine methyl ester (Leu-OMe) (Scheme 1).

The linear N-amide alkylated dipeptide methyl ester is then cyclized either in a mixture of DMF/piperidine [133], or under a microwave at 180 °C [134]. A similar procedure has been used to prepare a library of DKPs comprising cLP. In this case, the authors started with L- or D-Pro-OMe coupled to *N*-Boc-protected amino acids using carbodiimide-mediated conditions. The *N*-Boc group of the dipeptide was then cleaved under acidic conditions, prior to inducing the intramolecular cyclization upon treatment with piperidine at room temperature (Scheme 1a). The DKPs thus obtained were purified by column chromatography [133]. Ouzari and coworkers adapted this procedure to synthesize cyclo(L-Leu-L-Pro) using a methylated leucine and a Fmoc strategy. The standard coupling reagents EDC and HOBt provided the Fmoc-protected dipeptide. The ester was then saponified with aqueous LiOH, prior to performing the final cyclization in the presence of EDC/HOBt. After chromatography, the final product cLP was obtained with an overall yield of 50% (Scheme 1b). This procedure, using N-Fmoc-protected amino acids under standard EDC/HOBt-mediated conditions, was considered more efficient than the Boc strategy [58]. A slightly lower yield (42.6%) was reported in a recent study for the synthesis of cyclo(L-Pro-L-Leu) using the EDC/HOBt coupling procedure outlined in Scheme 1 [135].

It is worth underlining that the four cyclo(Leu-Pro) stereoisomers can epimerize, as represented in Figure 2 [71,136]. The trans isomer is generally favored, being more stable than the cis isomer [136]. The absolute configuration of the four isomers of cLP can be precisely determined by chiral gas chromatography [71] or using electronic circular dichroism (ECD) [134]. The isomer cyclo(L-Leu-L-Pro) seems to play a particularly important role in bacterial communications [71]. This *L-L* isomer of cLP is also more efficient at inhibiting conidia germination and at reducing leaf lesion size caused by the cucumber plant pathogen *C. orbiculare* [57].

An alternative process has been reported using L- or D-Pro and L- or D-Leu in the presence of Na trimetaphosphate (P3m) under alkaline aqueous condition (pH 11.7, 35 °C for 6 days). In this case, a clear preference for the formation of the cyclized product was observed when using D-Leu/Pro over L-Leu/Pro [138]. P3m is a convenient activator to produce dipeptides and Pro-containing DKPs from various amino acids (Scheme 1c) [137,139,140]. There are other options for synthesizing cLP and related DKPs [141,142]. Overall, the chemical synthesis of cLP poses no major difficulty, and the product can be produced in large quantities, if needed.

Chemo-enzymatic methods have also been considered for the synthesis of DKPs. A process based on the adenylation reaction of amino acids in the presence of the adenylation domain of tyrocidine synthetase A (TycA-A), followed by a cyclization, has been proposed. The method proved efficient for the synthesis of cyclo(L-Trp-L-Pro). It can be applied to other Pro-containing DKPs, like cLP [143]. The linkage of an adenylation domain is a convenient option to activate both L- and D-amino acids [144]. The DKP motif thus obtained can be used directly in pharmacological studies or exploited to synthesize longer peptides, notably via the use of Boc-DKP building blocks [145,146]. There are suitable procedures for integrating the DKP motif into peptides and in combinatorial chemistry [147,148,149].

## 4. Bioactivities of cLP

Multiple pharmacological activities have been reported for cLP, notably against a diversity of human pathogens. The different activities are discussed in turn below.

### 4.1. Antioxidant and Bioprotective Activities

Like other cyclic dipeptides, cyclo(L-Leu-L-Pro) can act as a scavenger of oxygen free radicals such as O_2_^−•^ and OH^•^. The product has been shown to inhibit OH^•^. But its efficiency was a little inferior to that of other DKPs, notably cyclo(L-Phe-L-Pro) [150]. The quenching of oxygen free radicals contributes to the bioprotective action of cLP, notably against food-borne pathogenic fungi [59]. This antioxidant effect is not specific to cLP. It has been evidenced with a range of cyclic peptides containing L-leucine and possessing polar amino acid residues, like Pro, but also Asp, Cys, Glu, Lys, Ser, and Trp [151]. However, cLP is an efficient scavenger of reactive oxygen species (ROS), much more effective than the corresponding linear dipeptide LP [152].

### 4.2. Antibacterial Activities

Many cyclic dipeptides exhibit broad spectrum antimicrobial effects. This is the case for cLP, which can inhibit the growth of pathogenic microorganisms, alone or in combination with other cyclic dipeptides. In particular, the combination of cyclo(L-Leu-L-Pro) and cyclo(L-Phe-L-Pro) showed a synergistic activity against the pathogen *Salmonella typhimurium*, responsible for enterocolitis [110]. cLP revealed also a prominent activity against the uropathogen *Serratia marcescens* and the Gram-positive pathogen *Staphylococcus epidermidis*, which is often associated with bone infections [153,154]. In the latter case, cLP showed a prominent antibiofilm efficacy, without bactericidal effects. The product markedly inhibited biofilm formation (64% and 82% inhibition at 128 and 256 μg/mL) without blatantly altering the basic cellular action of the bacteria. cLP affected the chemical integrity of the extracellular polymeric substance (EPS) matrix, with a notable reduction in the polysaccharide and protein components, and a reduction in the charge of secreted EPS [153]. An antibiofilm activity was also reported when using the Gram-positive food-borne pathogen *Listeria monocytogenes*. In this case, cLP was a bactericide (MIC = 512 µg/mL), but a marked antibiofilm activity was observed at non-bactericidal doses. It reduced the biofilm assemblage and the bacterial virulence [45]. Other examples of antibacterial activities observed with cLP are listed in Table 1. cLP itself or bacterial fractions containing cLP have shown activity against diverse microorganisms, including multidrug-resistant bacteria [78].

**Figure 4:**
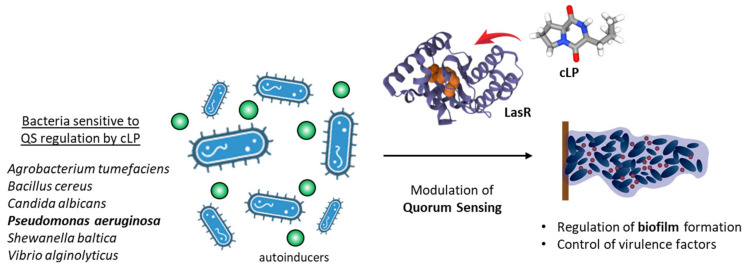
cLP participates in the regulation of bacterial communication through an inhibition of quorum-sensing (QS) and the control of autoinducers. cLP can form stable complexes with the QS regulator LasR. A model of *P. aeruginosa* LasR ligand-binding domain bound to its autoinducer (N-3-oxo-dodecanoyl-L-homoserine lactone) is shown (from PDB: 2UV0). cLP can repress expression of QS-related genes in *P. aeruginosa* and inhibit the production of QS-regulated virulence factors [89].

cLP plays a role in quorum-sensing (QS) signaling, which is a mechanism of bacterial regulation relying on the production, release, and detection of signaling molecules (autoinducers) (Figure 4). The QS system helps bacteria to communicate with each other in a density-dependent manner and plays a role in the regulation of pathogenicity. This mechanism has been well studied with proline-containing cyclodipeptides, notably with cyclo(L-Pro-L-Tyr) (also known as maculosin), which targets the LasR receptor [155]. A molecular modeling analysis suggested that cLP can form stable complexes with the QS protein LasR and suppress the synthesis of QS-associated virulence factors in *Pseudomonas aeruginosa* PAO1 [89]. It can also form stable complexes with other proteins with a role in QS, such as cAMP-dependent protein kinase regulators RAS1 and adenylate cyclase CYR1 in *Candida albicans* [156]. In the bacteria *Shewanella baltica*, which is a specific spoilage organism of fishes, cLP was shown to act as a QS signal acting through the central regulator of stress resistance RpoS, implicated in biofilm formation and quorum-sensing [157]. cLP was shown to activate QS biosensors of the plant pathogen *Agrobacterium tumefaciens*, as observed with cyclo(L-Pro-L-Tyr) [94]. The two dipeptides cyclo(L-Leu-L-Pro) (cLP) and cyclo(L-Phe-L-Pro) have been identified in an extract of *Vibrio alginolyticus* BC25, which showed a significant anti-QS activity [158]. cLP has been identified also in an extract from *Bacillus cereus* RC1, with a role as a QS regulator for the pathogen *Lelliottia amnigena*, which causes soft rot diseases in onions and potatoes [159]. With no doubt, cLP can be considered as a quorum-sensing regulator and thus as a molecular actor of the bacteria social network (Figure 4).

### 4.3. Anticariogenic Activity

The capacity of cLP to attenuate the biofilm formation and virulence of the bacterium *Streptococcus mutans* suggested the use of this product to combat dental caries. *S. mutans* is a major oral pathogen, and cyclic dipeptides are considered as effective inhibitors of the adherence of microorganisms to the dental surface [160]. cLP displays activities against different bacteria implicated in dental caries and periodontal diseases, primarily *S. mutans* [64], but also other bacteria such as *Streptococcus gordonii* implicated also in oral biofilm formation. In the oral bacterium *Veillonella tobetsuensis*, cLP was found to inhibit the development of *S. gordonii* biofilm, without the inhibition of planktonic cell growth [119,161]. At this level, a potential mechanism of action refers to the capacity of cLP to down-regulate critical virulence proteins related to the D-alanylation of lipoteichoic acid, which is a process contributing to biofilm formation and acidogenesis [162]. cLP has been shown to target the D-alanylation of lipoteichoic acid, inhibiting D-alanine biosynthesis and thereby the adherence of bacteria to the dental surface [163]. cLP emerges as a promising therapeutic agent in the management of oral infections.

### 4.4. Antifungal Activity

Cyclo(L-Leu-L-Pro) stands as a potent inhibitor of growth and aflatoxin production in fungi, but the mechanism at the origin of this effect is not precisely known. For example, cLP isolated from the Gram-negative opportunistic bacterium *Achromobacter xylosoxidans* was shown to inhibit the production of aflatoxins by the fungus *Aspergillus parsiticus* [62,164]. Cyclo(L-Leu-L-Pro) and isomer cyclo(D-Leu-D-Pro) showed comparable antifungal potency (IC_50_: 0.20 and 0.13 mg/mL, respectively) [62]. However, in another study comparing the antifungal activity of cLP isomers against the fungus *Colletotrichum orbiculare* responsible for anthracnose in cucumber plants, isomer cyclo(L-Leu-L-Pro) was more efficient than cyclo(D-Leu-D-Pro), and isomer cyclo(D-Leu-L-Pro) did not exhibit antifungal activity. In this specific case, cLP isomers showed a distinct biocontrol capacity, and the *LL* stereoisomer of cLP was preferred for disease control [57]. The different antifungal potencies of cyclo(L-Leu-L-Pro) and cyclo(L-Leu-D-Pro) have been underlined in another study: the isomer *LL* was found to be more potent against *Aspergillus flavus* and *Fusarium oxysporum*, whereas the isomer *LD* was more efficient than the *LL* isomer against *Candida albicans* [67]. The difference might be related to the different spatial configurations of the two products. The *LL* isomer has been shown to adopt a symmetric boat shape conformation, whereas the ring of the *DL* isomer is more planar [165].

**Figure 5:**
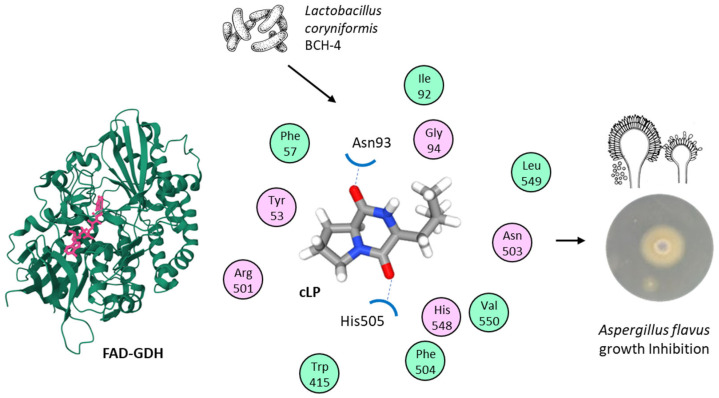
cLP from *Lactobacillus coryniformis* BCH-4 can bind to the active site of enzyme FAD-GDH and inhibits proliferation of *A. flavus*. On the left, a molecular model of FAD-GDH bound to dihydroflavine-adenine dinucleotide (FADH2 in pink) (PDB: 4TNT). The modeling analysis suggested that cLP can form stable complexes with FAD-GDH (flavin adenine dinucleotide-dependent glucose dehydrogenase), but also with dihydrofolate reductase and urate oxidase [168].

The fungal protein FAD-GDH has been proposed as a potential target for cLP. Flavin adenine dinucleotide (FAD)-dependent glucose dehydrogenase (GDH) is an oxygen-independent enzyme commonly found in fungi and considered as a potential target for antifungal agents [166,167]. A molecular modeling (docking) analysis revealed that cLP could form a stable complex with the FAD-GDH of *A. flavus* via an interaction with key residues (Asn93 and His505) in the enzyme active site (Figure 5) [168]. This hypothetical protein target for cLP remains to be validated experimentally. In fact, FAD-GDH is perhaps not the sole target implicated in the antifungal action of cLP. The related product cyclo(L-Ala-L-Pro) also inhibits aflatoxin production in aflatoxigenic fungi, and here the activity has been associated with the inhibition of *A. flavus* glutathione *S*-transferase (AfGST) [169]. It would be interesting to compare these two DKPs for their ability to inhibit AfGST.

cLP was found to be efficient at reducing the growth of *Fusarium culmorum* DMF 0109, which is a soil-borne fungal pathogen causing major damage in small-grain cereals like wheat. After 72 h, the mycelium growth on bread slices containing cLP at 10 mmol/kg was reduced by 83%, whereas the effect was limited to about 37% and 18% with cyclo(L-Phe-L-Pro) and cyclo(L-Tyr-L-Pro), respectively. This fungal strain turned out to be much more sensitive to cLP compared to other fungi, like *Penicillium chrysogenum* DBM 4062, which is also a contaminant possibly found on bread slices [135].

A significant activity of cyclo(L-Leu-L-Pro) against the fungus *Colletotrichum orbiculare* responsible for cucumber anthracnose has been reported. The cyclic dipeptide was found to reduce conidia germination, appressorium formation, and the occurrence of lesion in this species [95]. cLP isolated from the lactic acid-producing bacteria *Loigolactobacillus coryniformis* BCH-4 (isolated from rice) has been shown to produce antifungal metabolites, notably propanedioic/butanedioic acid derivatives active against *Aspergillus flavus* [168,170]. cLP and other proline-based cyclic dipeptides have been isolated also from this fungus.

### 4.5. Antiparasitic Activity

Gancidin W, isolated from the bacterial species *Streptomyces* sp. SUK 10, has been shown to reduce parasitemia in mice infected with *Plasmodium berghei* PZZ1/100, which is a quinine-sensitive and chloroquine-resistant strain responsible for malaria in rodent species. At the daily dose of 3.125 μg/kg, gancidin W inhibited the parasite growth by 78.5% and increased significantly the survival period of infected mice, without causing major toxic effects [39,171]. Gancidin W is responsible, at least in part, for the marked antiplasmodial effects observed upon the administration of an ethyl acetate extract of this bacterium SUK10 [38]. But this type of crude extract may contain different DKPs, notably Pro-based DKPs, which are known to display antiparasitic effects [172]. Interestingly, cyclo(L-Pro-L-Leu) has been shown to inhibit also the growth of *P. falciparum* strains 3D7 (drug-sensitive) and Dd2 (drug-resistant), whereas the three related DKPs cyclo(L-Pro-L-Phe), cyclo(L-Pro-L-Val), and cyclo(L-Pro-L-Tyr) showed no effects [116]. The unique antiplasmodial properties of cLP warrant further investigation.

An activity against the plant-parasitic nematode *Meloidogyne incognita* has been reported with cLP. The product increased the mortality rates of *M. incognita* second-stage juveniles (J2) in this species. It killed half of the population at the dose (LC_50_) of 65.3 μg/mL [93,96]. A similar nematocidal effect against the root-knot nematode (*M. incognita*) had been reported previously with the isomer cyclo(L-Leu-D-Pro) [44].

### 4.6. Anticancer Activity

**Figure 6:**
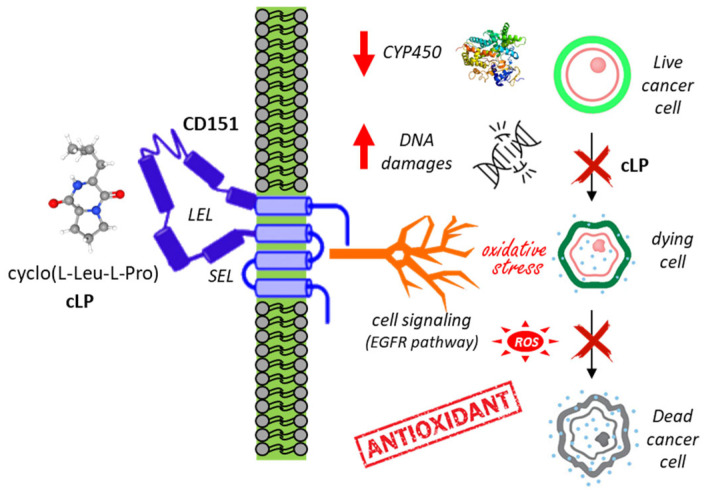
Through targeting the CD151-EGFR signaling pathway, cLP inhibits the increased expression of CD151 in MCF-12A epithelial breast cells exposed to the oxidative stress-inducer *tert*-butyl hydroperoxide (*t*BHP). The cLP-triggered down-regulation of CD151, reactive oxygen species (ROS), and cytochrome p450 contributes to reducing the oxidative stress [152,182].

The tetraspanin CD151 plays important roles in cell–cell communication and contributes to signal transduction, epithelial–mesenchymal transition (EMT), and other cellular processes [173,174]. It is considered as a potential drug target to combat several pathologies, including cancers and viral diseases [175,176]. Antibodies and small molecules targeting CD151 are researched in the treatment of these pathologies. Encouraging results have been obtained with anti-CD151 mAbs [177,178]. The selective targeting of this tetraspanin with a small molecule is more challenging, but a few compounds have been shown to interact with the large extracellular loop (LEL) of CD151, such as diallyl sulfide derivatives, 2-thio-6-azauridine, and pyrocatechol [179,180,181,182]. Remarkably, cLP has been shown also to interact with this integrin-binding protein so as to inhibit the growth and migration of solid tumor cells. Indeed, Malla and co-workers demonstrated that cLP inhibited the proliferation, cell cycle progression, and migration of triple-negative breast cancer (TNBC) cells, via a process implicating the down-regulation of CD151 from the cell surface. A molecular modeling analysis suggested that cLP could interact directly with the LEL of CD151 so as to perturb the CD151-EGFR signaling pathway [183]. A subsequent study by the same authors also concluded with the selective targeting of CD151 with cLP and the cLP-induced down-regulation of the tetraspanin associated with an anti-oxidative function in breast epithelial cells. Remarkably, the cyclic molecule cLP was shown to reduce cytochrome p450 expression levels, intracellular ROS, lactate dehydrogenase (LDH) release, and DNA damage in treated cells, whereas the linear dipeptide LP showed little effects [152] (Figure 6). CD151 is known to play a role in cancer progression through EGFR/ErbB2 signaling. An overexpression of CD151 promotes tumor proliferation, whereas a knockdown of CD151 inhibits tumor proliferation, migration, and invasion [184,185]. cLP displays caspase-dependent proapoptotic properties and inhibits the migration and invasion of cancer cells [52].

A few other studies have evidenced the antiproliferative action of cLP against cancer cells, notably when using TNCB cells such as the cell line MDA-MB-231 [103]. cLP was also shown to inhibit cell growth and to trigger the apoptosis of HT-29 colorectal cancer cells in vitro, and to inhibit tumor progression in a zebrafish model, but its effects were equivalent to those observed with three other DKPs such as cyclo (L-Val-L-Pro), cyclo(L-Phe-L-Pro), and cyclo(L-Tyr-L-Pro) [72].

## 5. Conclusions

Since the discovery of gancidins in the late 1950s and the formal identification of gancidin W from a strain of Gram-positive filamentous bacterium *Streptomyces gancidicus* twenty years later [32], this diketopiperazine derivative has been observed in a large diversity of microorganisms, notably in bacteria belonging to *Bacillus*, *Pseudomonas*, and *Streptomyces* genera. Gancidin W, better known as cyclo(L-Leu-L-Pro) (cLP), has been identified in about 60 bacterial species, generally in combination with other cyclic dipeptides and DKP derivatives (Table 1). cLP is a common natural product, easily accessible through extraction from natural sources or via chemical synthesis. Different chemical approaches to cLP have been proposed, with yields in the range of 45–50% [58,135]. This homodetic cyclic peptide composed from leucyl and prolyl residues presents different isomers (D/L), cis-cyclo(L-Leu-L-Pro) being the most important one. cLP is commercially available (at a cost of 400–800$/g).

At the pharmacological level, cLP emerges as an effective antimicrobial agent, notably efficient for limiting biofilm formation. These properties are not unique to cLP. Similar effects have been underlined with other proline-containing DKPs [132,186,187,188]. However, in some cases, effects have been demonstrated with cLP and not with related DKPs, such as the antiplasmodial activity observed with cyclo(L-Pro-L-Leu) but not with cyclo(L-Pro-L-Phe), cyclo(L-Pro-L-Val), and cyclo(L-Pro-L-Tyr) [116]. In the same vein, cyclo(L-Pro-L-Leu) turned out to be much more potent than cyclo(L-Pro-L-Phe), cyclo(L-Pro-L-Val), and cyclo(L-Pro-L-Tyr) at inhibiting mycelium growth of the phytopathogenic *Fusarium culmorum* strain [135]. cLP is a conventional DKP, but there is something special about it which makes the product particularly interesting. It is a potent antifungal and antiplasmodial agent, but additional studies are needed to better characterize its molecular targets and to better differentiate cLP vs. other DKPs.

**Figure 7:**
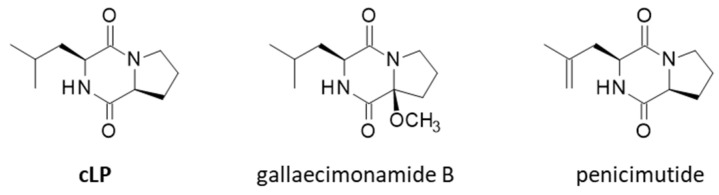
Structures of cLP and two related natural products.

Studies shall continue to further investigate its properties. It will help to better exploit the product and to comprehend the mechanisms of action of related natural substances. There are close analogs of cLP, such as penicimutide and gallaecimonamide B [88,189] (Figure 7). A better understanding of the mechanisms of action of cLP can help in defining and exploiting the properties of related products.

## Data Availability

No new data were created.
